# Diagnostic accuracy of GPs when using an early-intervention decision support system: a high-fidelity simulation

**DOI:** 10.3399/bjgp16X688417

**Published:** 2017-01-31

**Authors:** Olga Kostopoulou, Talya Porat, Derek Corrigan, Samhar Mahmoud, Brendan C Delaney

**Affiliations:** Department of Surgery and Cancer, Imperial College London, London.; Department of Primary Care and Public Health Sciences, King’s College London, London.; Health Research Board Centre for Primary Care Research, Royal College of Surgeons in Ireland, Dublin.; Department of Primary Care and Public Health Sciences, King’s College London, London.; Department of Surgery and Cancer, Imperial College London, London.

**Keywords:** decision support systems, diagnosis, diagnostic accuracy, diagnostic errors, electronic health record, first impressions, general practice

## Abstract

**Background:**

Observational and experimental studies of the diagnostic task have demonstrated the importance of the first hypotheses that come to mind for accurate diagnosis. A prototype decision support system (DSS) designed to support GPs’ first impressions has been integrated with a commercial electronic health record (EHR) system.

**Aim:**

To evaluate the prototype DSS in a high-fidelity simulation.

**Design and setting:**

Within-participant design: 34 GPs consulted with six standardised patients (actors) using their usual EHR. On a different day, GPs used the EHR with the integrated DSS to consult with six other patients, matched for difficulty and counterbalanced.

**Method:**

Entering the reason for encounter triggered the DSS, which provided a patient-specific list of potential diagnoses, and supported coding of symptoms during the consultation. At each consultation, GPs recorded their diagnosis and management. At the end, they completed a usability questionnaire. The actors completed a satisfaction questionnaire after each consultation.

**Results:**

There was an 8–9% absolute improvement in diagnostic accuracy when the DSS was used. This improvement was significant (odds ratio [OR] 1.41, 95% confidence interval [CI] = 1.13 to 1.77, *P*<0.01). There was no associated increase of investigations ordered or consultation length. GPs coded significantly more data when using the DSS (mean 12.35 with the DSS versus 1.64 without), and were generally satisfied with its usability. Patient satisfaction ratings were the same for consultations with and without the DSS.

**Conclusion:**

The DSS prototype was successfully employed in simulated consultations of high fidelity, with no measurable influences on patient satisfaction. The substantially increased data coding can operate as motivation for future DSS adoption.

## INTRODUCTION

A report by the National Academy of Medicine concluded that ‘most people will experience at least one diagnostic error in their lifetime, sometimes with devastating consequences’.^1^ In primary care, GPs have the difficult task of detecting potentially serious but uncommon disease among predominantly non-serious complaints. Being reflective or considering alternatives is common advice to guard against diagnostic error;^2^ however, timing is crucial. First impressions — that is, the first hypotheses that come to mind — can exert a strong influence on subsequent diagnosis and management decisions. For example, using a think-aloud methodology, Kostopoulou and colleagues found that, if GPs did not explicitly acknowledge the possibility of cancer after reading a short patient description and the presenting problem, they were significantly less likely to diagnose cancer at the end of the consultation and refer to a specialist.^3^ In two studies in the UK and Greece, Kostopoulou and colleagues also found that providing GPs with a list of diagnostic suggestions at the start of online simulated consultations was associated with increased diagnostic accuracy and better management, in comparison with unaided control.^4^^,^^5^ Taken together, these findings attest to the importance of the initial stage of hypothesis generation for the final outcome of the diagnostic process, and the importance of intervening as early as possible to influence this initial stage, before GPs embark on testing hypotheses.

As part of TRANSFoRm, a 5-year (2010 to 2015) European collaborative project (www.transformproject.eu), the authors designed and developed a prototype decision support system (DSS) for general practice, based on this principle of early support. It involves displaying a list of possible diagnoses as soon as the GP enters the reason for encounter (RfE), and before seeking further information. The prototype’s interface design was guided by prior elicitation of user decision requirements.^6^

**Figure 1.**
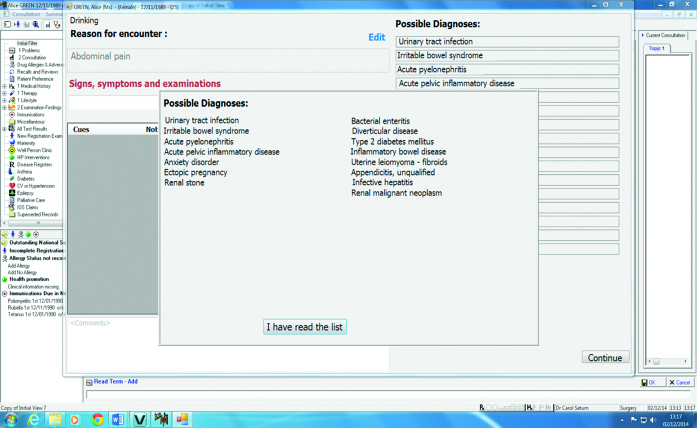
***Screenshot of the initial list of suggested diagnoses for a scenario. The list is displayed as soon as the GP enters a reason for encounter. In the screenshot here, the reason for encounter is ‘abdominal pain’.***

For its evaluation, the DSS prototype was fully integrated with a commercial electronic health record (EHR) system, Vision by In Practice Systems Ltd (www.inps.co.uk/vision). Appendix 1 provides further details. The prototype currently supports three RfEs: chest pain, abdominal pain, and dyspnoea. When the GP enters an RfE, a list of diagnostic suggestions appears, appropriate for the patient’s age, sex, and RfE (Figure 1). The diagnoses on the list are ordered according to published incidence rates: common (>50/100 000 per annum [p/a]), uncommon (10/100 000 to 50/100 000 p/a), and rare (<10/100 000 p/a). Within each category of incidence, the diagnoses are ordered randomly. Once the GP starts coding symptoms and signs into the DSS, the order of the displayed diagnoses changes dynamically to reflect both diagnostic incidence and the amount of diagnostic information accumulated during the consultation.

**How this fits in**

In previous research, the authors measured the importance of first diagnostic impressions for subsequent diagnosis and management. They have also formulated and tested the principle of early diagnostic support, providing GPs with a list of diagnostic suggestions based on the patient’s age, sex, risk factors, and presenting problem at the start of the consultation, before further information gathering. In two randomised controlled experiments in the UK and Greece, where GPs diagnosed and managed a series of simulated patients online, the authors found that GPs provided with early support gave more accurate diagnoses than unaided control. The study reported here demonstrates that a decision support system designed around the principle of early support, and integrated with the electronic health record, can be used by GPs in realistic consultations and can improve diagnostic accuracy without measurable influences on tests ordered, consultation length, or patient satisfaction.

**Figure 2.**
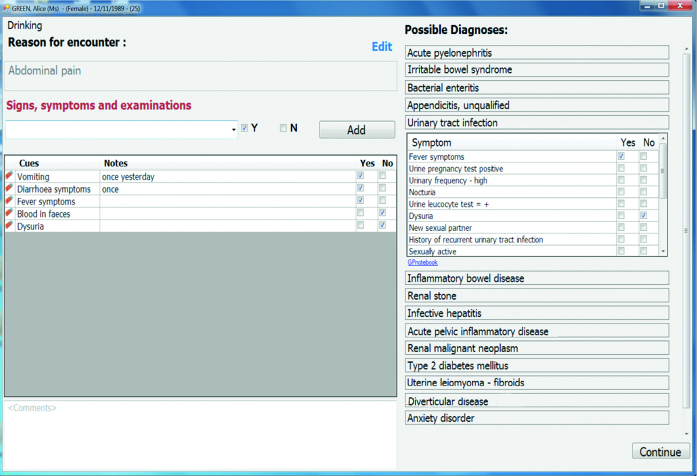
***Screenshot of the main consultation screen. On the left-hand side of the main consultation screen, coded information accumulates in the order in which it is entered. On the right-hand side, the list of diagnostic suggestions is visible. By clicking on a diagnosis, the user (GP) can view its associated symptoms and signs, and select their presence or absence.***

GPs can code symptoms and signs into the DSS in two ways:

- using a context-sensitive search box. For example, typing ‘vo’ when the RfE is abdominal pain will trigger suggestions that include ‘vomiting’; or
- selecting a suggested diagnosis from the list, which reveals the associated symptoms and signs (Figure 2).

In both cases, GPs can indicate the presence or absence of a symptom or sign by checking the relevant box. They can also add free-text notes next to each symptom or sign (Figure 2). All information coded during the consultation accumulates on the screen in the sequence it was entered. At the end of the consultation, the information is transferred automatically into the patient’s EHR, correctly coded, and structured (Appendix 1).

Full-scale clinical trials of DSS for diagnosis are complicated by the lack of well-defined measures of diagnostic performance applicable to real-world settings, and the infrequency of errors causing death or hospitalisation. Furthermore, the DSS is still at the prototype stage, only supporting three RfEs and without sufficient knowledge base for real-life use. Therefore, to evaluate the DSS at its current stage of development, the authors created a high-fidelity simulation of the clinical consultation that gave sufficient control (for example, by using pre-designed clinical scenarios), but was more similar to real life than consultations with computerised scenarios. The authors used actors as patients, a real EHR system, and a simulated GP surgery. The aim was to measure the prototype’s effectiveness, usability, and potential impact on the consultation and patient satisfaction.

## METHOD

### Materials

The authors employed 12 detailed patient scenarios, four for each RfE. Of these, 10 had been developed in previous studies of diagnosis in general practice, and depicted common diseases, such as angina and asthma, as well as less common ones, such as aortic stenosis and cancer.^3^^,^^4^ The remaining two were designed to be entirely straightforward for the present study, thus increasing the similarity with the range of cases seen in general practice. The complete scenario information allowed for a single correct diagnosis. Patient age ranged from 22 to 70 years. Half of the patients were male.

In the absence of sufficiently robust clinical data to derive a comprehensive list of differential diagnoses, two academic GPs independently generated lists of diagnostic suggestions for each scenario. They then reviewed their lists and produced an agreed list for each scenario. The authors entered each patient’s age, sex, risk factors, and RfE — but no other symptoms or signs — into two commercial differential diagnosis generators that were found to produce more accurate diagnostic lists in a comparison with other similar tools^7^: DXplain (dxplain.org) and Isabel (isabelhealthcare.com). The resulting lists were merged with the GPs’ agreed list. Finally, a third GP reviewed the merged list for plausibility and duplication. The lists included between 16 and 22 diagnoses, depending on RfE, patient age, and sex.

### Design

The study employed a within-participant design. Each GP took part in two sessions separated by at least 1 day. In the first session, the authors measured baseline performance. GPs consulted with six standardised patients (actors trained in the assessment of medical communication skills), using their usual EHR system, Vision, without the DSS prototype. GPs were free to use the EHR as they wished, coding and adding text as in their usual practice. In the second session, the GPs consulted with six different standardised patients (referred to henceforth as patients) using the DSS integrated with Vision. The two sessions were matched for difficulty based on the per cent accuracy (correct diagnoses divided by all diagnoses) obtained in the two previous studies.^3^^,^^4^ Specifically, 0.54% average accuracy was obtained across the six patients of one session, and 0.52% across the six patients of the other session. The order of patient presentation was randomised within each session. Counterbalancing sessions across participants ensured that each patient was seen with and without the DSS equally frequently. The DSS prototype was always used at the second session, to ensure that experience with it would not influence baseline performance. A potential training effect that could enhance performance in the second session, and thus confound the effect of the DSS, was accounted for in the statistical analyses.

### Procedure

The study took place at King’s College London, in a room set up to resemble a typical consultation room. The patients’ demographics and past medical history were pre-loaded onto the EHR. Appointments were organised in 10-minute slots, and GPs could see on their screen if they were running late. At the start of the DSS session, GPs received brief training on the DSS. GPs could not examine the patient, but could indicate what physical examinations they wished to perform, and the patient gave them the findings promptly. If they ordered investigations that did not require specialist referral, they received the results at the end of the consultation. At the end of each consultation, GPs recorded their working diagnosis, certainty (from 0 ‘not at all certain’ to 10 ‘absolutely certain’), and any differential diagnoses. They then chose one or more from a list of management options: prescribe, refer to specialist or for specialist tests, arrange follow-up appointment, or ask the patient to come back if symptoms persist. These were specified further as, for example, refer for what, which specialist, what type of referral, and so on. Vision automatically recorded the length of time that the patient record remained open. After each consultation the patients filled in a standardised consultation satisfaction questionnaire (CSQ), which evaluates four dimensions: general satisfaction, professional care, depth of relationship, and length of consultation.^8^

At the end of each study session, the GPs indicated how realistic they found the consultations (from 0 ‘not realistic at all’ to 5 ‘like real life’) and how representative of general practice the scenarios were (from 0 ‘not representative at all’ to 5 ‘very representative’).

Finally, at the end of the DSS session, GPs completed the post-study system usability questionnaire (PSSUQ), which evaluates four dimensions: overall satisfaction, system usefulness, information quality, and interface quality.^9^

### Sample size

A previous study obtained a 6% absolute difference in diagnostic accuracy using nine patient scenarios: 63% (control group) versus 69% (early support group).^4^ The intraclass correlation *ρ* was 0.05 and significant. To detect the same absolute difference in a paired-samples comparison of proportions (α = 0.05 and 90% power) would require 26 GPs. The authors adjusted this number by the design effect expected for a cluster of six patients per session, using the formula DE = 1 + (*n* – 1) *ρ*, where *n* is the cluster size, and *ρ* the intraclass correlation of the previous study.^10^ The authors then adjusted the previously estimated sample size of 26 GPs by the design effect (1.25), which resulted in a minimum required sample size of 33 GPs (26 × 1.25 = 33).

### Recruitment

The authors sought to recruit fully qualified GPs, current or recent (within 1 year) users of the Vision EHR system. The National Institute for Health Research (NIHR) Primary Care Research Network (PCRN) contacted practices on the authors’ behalf in five local trusts (Bexley, Croydon, Greenwich, Kingston, and Richmond). The authors also posted a message on a closed Facebook group for UK GPs only. Participants were offered recompense at the standard clinical hourly rate. GPs interested in participating contacted the research team, who sent them the information sheet to read before deciding. All those who contacted the team agreed to participate. GPs were recruited on a first-response basis, until the required sample size was reached.

### Analyses

The primary outcome was diagnostic accuracy. Two measures were developed — a strict one based on the working diagnosis, and an inclusive measure based on both the working diagnosis and the differential. Responses were scored as either correct (1) or incorrect (0), depending on whether they included the correct diagnosis for the scenario. Management was scored as either appropriate (1) or inappropriate (0), based on whether patient harm could result from misdiagnosis or delay.

The authors measured the effect of DSS (versus baseline) on diagnostic accuracy and management using logistic regressions. To check whether performance improved over time, the authors regressed diagnostic accuracy on scenario order (1–6) separately for the baseline and DSS sessions. An improvement in diagnostic accuracy in the later scenarios of each session would indicate a training effect. Finally, linear regression was used to measure the effect of the DSS (versus baseline) on the following measures: number of investigations ordered, number of data items coded into the EHR, diagnostic certainty, and patient satisfaction (measured with the consultation satisfaction questionnaire).

All regression models were multilevel, with random intercept to account for clustered data within participants (12 scenarios per GP), and scenario order (1 to 12) as a repeated measure. The regression analyses were conducted using xtgee (Generalised Estimating Equations) on the Panel Data setup in Stata (version 13.1).

## RESULTS

Of the 34 GPs from Greater London who participated, half were male and 22 (65%) were recruited via the PCRN. The remaining 12 were recruited via Facebook. The sample’s average experience in general practice was 12.7 years (standard deviation [SD] 12.6, median 9.5, range 1 month to 40 years). Each data collection session lasted 2.5–3.5 hours. The DSS session took place on average 11 days after the baseline session (range 1–35 days, median 7 days). It took 20–40 minutes for the GPs to learn to use the DSS. Table 1 presents a summary of the results from the comparison between baseline and DSS sessions for all outcome measures.

**Table 1.** Summary table of results by study session^a^

**Outcome measure**	**Baseline session**	**DSS session**	**Odds ratios and regression coefficients (95% CIs)**
Diagnostic accuracy *(working diagnosis)*, %	49.5 (101/204)	58.3 (119/204)	OR 1.41 (1.13 to 1.77) *Z*= 2.98, *P*<0.003)
Diagnostic accuracy *(working diagnosis and differential)*, %	58.8 (120/204)	67.6 (138/204)	OR 1.50 (1.14 to 1.99) *Z*= 2.87, *P*<0.004
Appropriate management, %	59.3 (121/204)	66.2 (135/204)	OR 1.34 (1.01 to 1.78) *Z*= 2.06, *P*<0.004
Diagnostic certainty (0 to 10 VAS) Mean (SD)	7.61 (1.77)	8.01 (1.37)	B = 0.39 (0.12 to 0.67) *Z* = 2.80, *P*<0.005
Symptoms/signs coded into the EHR, mean (SD)	1.64 (1.96)	12.35 (6.34)	B *=* 10.71 (9.06 to 12.35) *Z*= 12.77, *P*<0.001
Tests ordered, mean (SD)	2.51 (2.96)	2.83 (2.92)	B =0.33 (−0.54 to 1.20) *Z*= 0.74, *P*<0.46
Consultation length, minutes, mean (SD)	13.73 (4.81)	14.42 (5.28)	B =0.69 (−0.28 to 1.67) *Z*= 1.39, *P*<0.17
Patient satisfaction, grand mean across all CSQ dimensions and SD	3.26 (0.70)	3.26 (0.63)	B =0.001 (−0.18 to 0.18) *Z*= 0.02, *P*<0.99

a *Dichotomous outcome measures are presented as percentages (number of correct responses out of 204 total responses per session). Continuous outcomes are presented as means (SD). Odds ratios, regression coefficients (B),* Z*-tests, and* P*-values from the regression analyses are also presented. CSQ = consultation satisfaction questionnaire. EHR = electronic health record. OR = odds ratio. SD = standard deviation. VAS = visual analogue scale.*

The absolute improvement in diagnostic accuracy with the DSS was 8% for the strict measure (49.5% at baseline versus 58% with the DSS) and 9% for the inclusive measure (59% at baseline versus 68% with the DSS). This improvement was statistically significant (odds ratio [OR] 1.41, 95% confidence interval [CI] = 1.13 to 1.77, *P* = 0.003), as were the improvements in diagnostic certainty and management (Table 1). The authors did not detect any improvement in accuracy over time, either at the baseline session (OR 1.10, 95% CI = 0.94 to 1.28) or the DSS session (OR 1.13, 95% CI = 0.96 to 1.32). It can therefore be concluded that the improvement obtained in the DSS session, which always came second, was due to DSS use alone rather than practising with more patient cases. Neither the number of investigations nor the length of consultation differed significantly between the baseline and DSS sessions (Table 1).

At baseline, 73.5% of the participants recorded information into the EHR only at the end of the consultation. In contrast, when using the DSS, GPs had to code information during the consultation in order for the initial list of suggested diagnoses to be updated. This resulted in the EHR being populated with considerably more coded information than at baseline. The actors’ satisfaction ratings were similar at both baseline and DSS sessions, for all four dimensions of the CSQ: mean ratings for general satisfaction 3.31 versus 3.29, professional care 3.45 versus 3.34, depth of relationship 2.82 versus 2.81, and length of consultation 3.56 versus 3.64 for baseline and DSS sessions, respectively.

GPs were generally satisfied with the usability of the DSS. Mean ratings on the PSSUQ were >4 (midpoint of the 1–7 rating scale) for all four dimensions: system usefulness 4.57 (SD 1.24), information quality 4.74 (SD 1.10), interface quality 4.73 (SD 1.34), and overall satisfaction 4.64 (SD 1.13).

GPs indicated that they found the consultations realistic, with mean ratings of 3.76 (SD 0.53) at baseline, and 4.01 (SD 0.67) with the DSS (from 0 ‘not realistic at all’ to 5 ‘like real life’). They also found the clinical scenarios representative of the patients that they see in their everyday practice, with mean ratings of 4.13 (SD 0.93) at baseline, and 4.23 (SD 0.82) with the DSS (from 0 ‘not representative at all’ to 5 ‘very representative’).

Nine GPs commented that, although the scenarios were representative, they would not expect to see them all in a single practice session (referring to the severity or urgency of the patient’s condition).

## DISCUSSION

### Summary

The authors developed a prototype DSS for diagnosis in general practice. At the heart of the prototype is the principle of early support, designed and tested in previous studies.^4^^,^^5^ The prototype was integrated with a commercial EHR system, and evaluated in a high-fidelity simulated environment. The authors found an 8–9% absolute improvement in diagnostic accuracy, which could translate into significant benefits to patients and healthcare systems, given the sheer number of primary care consultations. The improvement may appear small, but compares favourably with other diagnostic support systems, as the authors have outlined in a previous study.^4^ Using the DSS did not result in significant increases in consultation length and test ordering. This is especially important, given that GPs are reluctant to use a DSS if they worry that it is time consuming and leads to more tests.^11^

The GPs learned to use the DSS quickly, and found it usable. The patients did not perceive the GP and the consultation differently when the DSS was used. Thus, the authors found no evidence to support the traditionally expressed concerns by GPs, patients, and researchers about the impact on the doctor–patient relationship of using a DSS.^12^^–^14

Using the DSS resulted in, on average, 12 times more data coded into the EHR during the consultation. When using their EHR alone, most GPs recorded information only at the end of the consultation, after the patient had left the room. This practice results in a potentially biased EHR, where GPs record information consistent with their final diagnosis, due to biased memory or need for justification.^15^^,^^16^ It also diminishes the opportunity to enrich the evidence base via epidemiological research on routine data.

A diagnostic DSS integrated with the EHR, and with an interface that facilitates and encourages coding in real time, has the potential to become an instrument of change in everyday clinical practice and drastically transform the wealth of evidence obtained to create a learning health system for diagnosis.^17^

By facilitating coding, the DSS is likely to be more acceptable to users, given that it adds value to clinical practice through more complete records.

### Strengths and limitations

The scenarios ranged widely in diagnostic difficulty and GPs found them realistic and similar to those seen in practice. Nevertheless, some commented that the concentration of disease severity would be unlikely in a single practice session. If GPs had the impression that all patients had a serious problem, they may have changed their approach and adopted a more analytical style of consulting. This would have influenced their performance in baseline and DSS sessions, and possibly reduced the difference in diagnostic accuracy between sessions.

Although physically similar and with time limitations, the simulated environment did not contain the stresses and interruptions of a busy general practice. In real life, or when dealing with unannounced standardised patients,^18^ GPs may adopt a different approach to diagnostic problems, whether using the DSS or not. The usability, usefulness, and impact of the DSS in real practice thus remain to be tested. Nevertheless, a positive result in a high-fidelity simulation is an important step in technological evaluation, and the final precursor to large-scale clinical trials.

### Comparison with existing literature

Diagnostic decision support systems have not been enthusiastically adopted into routine clinical practice.^19^ Possible reasons include lack of high-quality diagnostic data, lack of integration with the EHR and the physician’s workflow, not following human factors principles, support provided too late in the consultation, and lack of physician acceptance or perceived need.^1^^,^^19^^–^21

This DSS goes a considerable way towards addressing these problems, and has the potential to be employed successfully, and lead to improved coding, diagnosis, and management, without significant costs in time, tests, and patient satisfaction.

### Implications for research and practice

Cognitive interventions to reduce diagnostic error^2^ typically do not specify or pay attention to the best timing for intervention. In primary care, on the basis of these findings, the authors recommend interventions supporting the earliest possible stages of the diagnostic process. Designing such interventions into decision support systems that have additional benefits, such as facilitating coding, is likely to increase acceptance.

The precise psychological mechanisms through which the DSS works, and its impact on cognition, are not known. It is possible that it has a debiasing effect by disrupting intuitive thinking based on first impressions, and encourages a more reflective or cautious approach. The generalisability of this effect in situations different from those used in this study, and its transferability to unaided diagnosis, are avenues for future research. Furthermore, there may be other ways of disrupting ‘System 1’ thinking,^22^ for example, by getting GPs first to generate their own differential, or by priming concepts of risk, uncertainty, or cautiousness at the very start of the diagnostic task.

In order to develop a functioning DSS for real-life evaluation, it is necessary to gather sufficient diagnostic data in the episode-of-care model^23^ (Appendix 1) to support a range of common RfEs,^24^ and calculate associations for at least moderately rare conditions, such as common cancers. Supporting decisions for more RfEs could be accomplished gradually, while using the system for data capture only. Therefore, it is possible to establish cycles of continuous learning and improvement, using a DSS integrated into a wide range of existing EHR systems.

## Appendix 1. Data capture and process

Integrating decision support systems’ (DSS) functionality into the electronic health record (EHR), the authors have developed methods for enhancing capture of coded diagnostic data within the consultation, and demonstrated methods of mining these data to produce diagnostic evidence.^1^^,^^2^

The Vision EHR supports Lawrence Weed’s episode-of-care model that can contain multiple consultations, linking a presenting problem (termed reason for encounter [RfE]) with the final diagnosis, as it has evolved during an episode of care (EoC).^3^ The authors expressed the relationships between RfE, diagnostic cues, and diagnoses as an ontology, that is, a computable statement of relationships between concepts.^1^ Specific data instances, expressed as Read codes (Clinical Terms v3), were bound to the relevant concepts and made available via the internet.^2^ The ontology is capable of supporting categorical associations, numerical predictive algorithms, and full Bayesian rules. It allows forward and backward reasoning of the rules, from diagnosis to cues (symptoms and signs), and from the RfE to diagnoses and cues. The data in the ontology can be curated manually from published literature or through data mining of suitable clinical data sources. To populate the diagnostic evidence base for the purposes of the evaluation study, the authors conducted systematic reviews of the clinical literature to identify the diagnostic cues associated with each suggested diagnosis in each scenario. They systematically searched Embase, PubMed, JAMA Evidence Reviews, Cochrane Medical Reviews, BMJ Clinical Evidence, NHS Clinical Knowledge Summaries, National Institute for Health and Care Excellence (NICE) guidelines, and Scottish Intercollegiate Guidelines Network (SIGN) guidelines.

To update the order of suggested diagnoses during the consultation, a dynamically updated cue count is maintained for each diagnosis, indicating the number of evidence cues (symptoms and signs) that are confirmed as present, based on the information that the GP enters into the DSS. When a relevant cue is added, the cue counts are re-calculated. The differential diagnoses are then re-ranked in descending order based on the cue count, within their incidence group (common, uncommon, or rare).

The integration of the DSS prototype with Vision was achieved by using a tool that communicates with the EHR through the EHR’s application programming interface (API — part of a computer system that is designed to allow interaction with components built by other developers), drives a window for data entry and display, and receives and sends data to/from the ontology via the internet. The prototype is brought up in an overlaying window by clicking an icon in the main Vision system. This approach is portable to any other EHR system, either through an API or by equivalent means, without having to alter the basic design of the EHR. At the end of a clinical consultation, all the information that the physician has coded into the DSS is transferred automatically into the patient’s EHR via its API, in the native coding and data structure of the EHR system.
